# Acute viral bronchiolitis as a cause of pediatric acute respiratory distress syndrome

**DOI:** 10.1007/s00431-020-03852-9

**Published:** 2020-11-07

**Authors:** Marwa M. H. Ghazaly, Nagla H. Abu Faddan, Duaa M. Raafat, Nagwa A. Mohammed, Simon Nadel

**Affiliations:** 1 Department of Pediatrics, Children University Hospital, Faculty of Medicine, Assiut University, Assiut, Egypt; 2 Paediatric Intensive Care Unit, St Mary’s Hospital, Imperial College London Healthcare NHS Trust, London, UK

**Keywords:** Bronchiolitis, Children, ARDS, Intensive care, Respiratory syncytial virus, RSV

## Abstract

The Pediatric Acute Lung Injury Consensus Conference (PALICC) published pediatric-specific guidelines for the definition, management, and research in pediatric acute respiratory distress syndrome (PARDS). Acute viral bronchiolitis (AVB) remains one of the leading causes of admission to PICU. Respiratory syncytial virus (RSV) is the most common cause of AVB. We aimed to evaluate the incidence of PARDS in AVB and identify the risk of RSV as a trigger pathogen for PARDS. This study is a retrospective single-center observational cohort study including children < 2 years of age admitted to the pediatric intensive care unit at St Mary’s Hospital, London, and presented with AVB in 3 years (2016–2018). Clinical and demographic data was collected; PALICC criteria were applied to define PARDS. Data was expressed as median (IQR range); non-parametric tests were used. In this study, 144 infants with acute viral bronchiolitis were admitted to PICU in the study period. Thirty-nine infants fulfilled criteria of PARDS with RSV as the most common virus identified. Bacterial infection was identified as a risk factor for development of PARDS in infants with AVB.

*Conclusion*: AVB is an important cause of PARDS in infants. RSV is associated with a higher risk of PARDS in AVB. Bacterial co-infection is a significant risk factor for development of PARDS in AVB.

**Table:** 

**What is Known:** • *Bronchiolitis is a common cause of respiratory failure in children under 2 years.* • *ARDS is a common cause of PICU admission.*	
**What is New:** • *Evaluation of bronchiolitis as a cause of PARDS according to the PALLIC criteria.* • *Evaluation of different viruses’ outcome in PARDS especially RSV as a commonest cause of AVB.*

**Supplementary Information:**

The online version contains supplementary material available at 10.1007/s00431-020-03852-9.

## Introduction

Acute respiratory distress syndrome (ARDS) is an acute lung injury that can be triggered by a heterogeneous set of pulmonary (direct lung injury) and extrapulmonary (indirect ling injury) etiologies. ARDS manifests as pulmonary inflammation, alveolar edema, and hypoxemic respiratory failure [1, 2].

ARDS in children (pediatric ARDS—PARDS) has been shown to have a lower mortality compared to adults [3, 4]. Furthermore, infections account for more than half of the cases of PARDS, particularly lower respiratory tract infections such as pneumonia and bronchiolitis, with viruses frequently implicated [3]. However, the importance of viral infections as a cause of PARDS in infants has not previously been described [5, 6].

About 3.5 million children under 5 years of age are admitted annually to hospitals due to lower respiratory tract infection (LRTI) caused by respiratory syncytial virus (RSV) worldwide [7]. RSV accounts for 22% of all acute LRTIs in children and is responsible for 66,000–199,000 deaths worldwide. Most childhood deaths due to RSV infection occur in developing countries [7].

The Pediatric Acute Lung Injury Consensus Conference (PALICC) definition of PARDS was developed to overcome the limitations of various definitions of ARDS, which were primarily designed and validated for adults (e.g., the Berlin definition of ARDS) [8].

The incidence of PARDS due to viral infection is still unknown [6]. RSV and rhinovirus are the most common respiratory pathogens in children under the age of 1 year and account for many admissions to pediatric intensive care units (PICUs) [9]. Despite this, few studies have investigated the frequency of viral-induced PARDS in infants and its impact on prognosis [4, 5, 10, 11].

## Aim

Our study aimed to evaluate the prevalence of PARDS, based on the recent PALICC definitions, in children with acute viral bronchiolitis (AVB) admitted to the PICU. We also aimed to compare patient characteristics and outcomes of PARDS with infection due to different viruses as a cause of AVB.

## Methodology

### Study design

We carried out a retrospective, descriptive, observational study that included children aged under 2 years admitted to the PICU at St Mary’s Hospital, London, who presented with acute respiratory failure due to AVB between January 2016 and June 2018.

Patients were classified into two groups: PARDS group, patients with a diagnosis of AVB and met the PALICC criteria for PARDS (Supplementary Table S1), and non-PARDS group, patients with a diagnosis of AVB who did not meet the PALICC criteria for PARDS.

We excluded patients with pre-existing lung disease or significant intra-cardiac shunt diagnosed by echocardiographic evaluation. We also excluded patients with missing records.

### Data collection

Patients were identified using the electronic database of PICU admissions (Careview, Phillips, UK). Inclusion criteria were children aged < 24 months with a recorded diagnosis of bronchiolitis using the SNOMED diagnostic code [12].

Collected data included patient demographics (age, sex, weight, race, ethnicity), comorbidities, daily recording of type, mode and parameters of ventilation, chest imaging, blood gas, pulse oximetry data, duration of mechanical ventilation, length of stay on PICU, and mortality.

### Assessment of etiology

All children had standardized sample collection on admission to PICU, which included nasopharyngeal aspirate (NPA) for viral polymerase chain reaction (PCR), blood culture, endotracheal tube (ETT) aspirate in intubated patients for viral PCR and bacterial culture, and other relevant cultures depending on clinical presentation. All samples were collected and analyzed using a standardized protocol.

The NPA and ETT aspirate samples were routinely tested for the following viruses using real-time multiplex PCR: RSV; rhinovirus; adenovirus; parainfluenza viruses 1, 2, and 3; enterovirus; and influenza virus A and B. Extended analyses were conducted on NPA or ETT specimens depending on advice from the Infectious Diseases Service. The results of all other bacterial cultures on respiratory specimens, urine, blood, or other cultures were also recorded. Some infants had similar diagnostic samples taken at their local hospital before transfer to PICU.

All patients also had routine hematology and biochemistry samples taken on admission to PICU.

### Outcomes

The primary outcome of this study was the development of PARDS according to PALICC criteria. Other outcomes recorded included the duration of mechanical ventilation (MV) and length of stay (LOS) in the PICU. In patients who required re-intubation after the first extubation, the time of first extubation was used for analysis.

## Statistical analysis

Statistical analysis was done using SPSS version 20.0 (SPSS Inc., Chicago, Illinois, USA). Statistical analysis for the comparison of patient criteria, clinical course, and outcomes among patients PARDS, RSV included Fisher's exact and Mann-Whitney tests, as appropriate with significance set at a *P*-value of <0.05. Univariate analysis was done first to identify potential risk factors and we added some factors as age ,wt., comorbidity ,RSV infection and bacterial coinfection them binary logistic regression analysis for development of PARDS as dependent and other factors as independent factors (age, comorbidity , rsv infection, bacterial coinfection).

## Results

Between January 2016 and June 2018, 144 children < 2 years of age were admitted to the PICU with a diagnosis of AVB.

Thirty-nine patients (27%) fulfilled the criteria of PARDS according to PALICC criteria. Median age for patients with PARDS was 60 days (IQR 35–172.5 days), 66% (*n* = 26) were male, 44% (*n* = 17) presented with apnea, 49% (*n* = 19) were premature, and 44% (*n* = 17) had previous comorbidities. There was no significant difference in baseline demographic characteristics in infants with and without PARDS (see Table 1).

**Table 1:** A comparison of demographics and clinical characteristics of all bronchiolitis infants with PARDS vs those without PARDS

	All (*n* = 144)	PARDS (*n* = 39)	No PARDS (*n* = 105)	*p* Value
Age (days), median (IQR)	56 (28–180)	60 (35–173)	56 (28–150)	0.4
Weight, kg, median (IQR)	4.6 (3–6.5)	4.4 (3–6.4)	5.1 (3.6–6.9)	0.09
Male gender	89 (66%)	28	61	0.2
Prematurity	62 (44%)	19	43	0.3
Comorbidity	43 (33.4%)	17	26	0.2
Respiratory comorbidity	16 (37%)	8	10	0.1
Apnea	70 (48.6%)	19	51	0.4
PIM-2r %	0.9 (0.6–1.7)	1.2 (0.6–1.7)	0.97 (0.7–1.2)	0.08
Caucasian	59	20	39	0.06

Positive viral PCR results were reported in 90% of PARDS cases, with RSV the most common pathogen (found in 51%), followed by rhinovirus (28%), human metapneumovirus (HMPV) (13%), and co-infection with more than one virus (18%). Bacterial co-infection was reported in 31% of cases (Table 2).

**Table 2:** Microbiological results and clinical outcomes of all bronchiolitis patients comparing patients with PARDS and without PARDS

	All (*n* = 144)	PARDS (*n* = 39)	No PARDS (*n* = 105)	*p* Value
RSV	68	20	48	*0.5*
Rhinovirus	28	6	22	*0.63*
HMPV	9	5	4	*0.06*
Adenovirus	3	2	1	*0.17*
influenza	6	1	5	*1*
Viral co-infection	23	6	17	0.06
Bacterial co-infection	22	12	10	*0.002*
Ventilation days	5.25 (3–8)	8	4.5	*0.0001*
PICU LOS	6.5 (5–10)	9.8 (5.6–10.8)	5.8 (2.7–6.1)	*0.0001*

*PARDS* pediatric acute respiratory distress syndrome, *RSV* respiratory syncytial virus, *HMPV* human metapneumovirus, *PICU* pediatric intensive care unit, *LOS* length of stay; categories were compared with Fisher exact test, medians were compared by Mann-Whitney test; significant results with *P *value < 0.05

All cases of PARDS required invasive ventilation. Twenty-five patients required escalation to high frequency oscillating ventilation (HFOV).

Our usual policy is to commence patients with respiratory failure who are admitted to PICU on parenteral antibiotics. Four children also received steroids as part of PARDS management. Two children died from their illness.

In regard of severity of PARDS, 17 cases were mild, 10 cases were moderate, and 12 were severe. Table 1 describes the patient factors associated with a diagnosis of PARDS.

## PARDS associated with RSV

Seventy-eight children had RSV as the cause of AVB, and RSV was the most commonly identified pathogen. Twenty of these cases fulfilled PARDS criteria.

Table 3 compares patient characteristics to differentiate between RSV positive PARDS and RSV negative PARDS.

**Table 3:** Demographics and clinical characteristics of patients with RSV PARDS, HPMV PARDS and other viruses PARDS group

	RSV with PARDS (*n* = 20)	HMPV with PARDS (*n* = 5)	Other viruses PARDS (*n* = 14)	*p* Value
Age, days, median (IQR)	66 (36–95)	127 (56–180)	168 (118–226)	0.002
Weight, kg, median	3.7 (3–4.7)	4 (3.8–5)	5.2 (4.7–5.8)	0.04
Male gender,	15	3	6	0.7
Prematurity	9	3	4	0.2
Comorbidity	8	2	4	0.5
Apnea	13	2	2	0.3
Caucasian	12	2	6	0.4
Co-infection	5	2	7	0.1
MAP (cm H_2_O)	12 (11–14)	14 (11–16)	13 (11–15)	0.2
FiO_2_ (%)	50 (44–60)	60 (40–80)	57 (48–66)	0.03
PEEP (cm H_2_O)	6 (5–7)	8 (5–8)	7 (5–8)	0.1
Ventilation days	9 (7.6–10.5)	16.2 (6–25)	10.2 (8–12.5)	0.01
PICU LOS days	11.7 (9.6–13)	17.5 (9.1–25)	11.5 (10–14)	0.0.02

*PARDS* pediatric acute respiratory distress syndrome, *RSV* respiratory syncytial virus, *IQR* interquartile range, *MAP* mean airway pressure, *FIO2* fraction inhaled oxygen, *PEEP* positive end expiratory pressure, *PICU* pediatric intensive care unit, *LOS* length of stay; categories were compared with Fisher exact test, medians were compared by Mann-Whitney test

Infants with RSV were younger and weighed less than infants with other viral etiologies. Infants with HMPV had more ventilation days and PICU stay (*p* = 0.02), (Table 4).

**Table 4:** Demographics and baseline characteristics of infants with PARDS stratified by individual viruses

	RSV (*n* = 15)	Rhino (*n* = 6)	Adeno (*n* = 3)	HMPV (*n* = 5)	Influenza (*n* = 1)	RSV/others (*n* = 5)
Age in days	35 (30–270)	210 (90–250)	285 (277.5–293)	127 (56–180)	28	120 (35–180)
Weight	3.15 (3.5–7)	6.3 (5.5–7)	7.5 (6.5–8)	4 (3.8–5)	2.2	4.6 (3–7.5)
Male	12	5	1	3	0	3
Prematurity	6	3	0	3	1	3
Comorbidity	6	4	0	2	0	2
Apnea	10	1	0	2	1	3
OI	10.6	13.5	14	13.3	10.9	7.5
PICU LOS	9 (7.7–18)	12 (6–18)	6.2 (5–10.5)	17.5 (9.1–25)	11	8.1 (6.8–8.3)
PARDS severity						
Mild	10	3	1	2	0	4
Moderate	4	0	1	1	1	1
Severe	1	3	1	2	0	0

## Risk factors for PARDS in acute viral bronchiolitis

Because the meaningfulness of logistic regression modeling we were restricted by the absolute number of PARDS cases, our model included four variables to evaluate the associations with the development of PARDS, choosing (RSV or not), age, bacterial coinfection, comorbidity. The presence of bacterial co-infection was significantly associated with the development of PARDS (OR = 1.9, *p* = 0.02) (confidence interval: 1.19–5.68).

## Discussion

This study reports one unit’s experience of the incidence of PARDS in children with AVB. We describe the frequency of PARDS in this cohort and demonstrate that specific viruses are associated with different outcomes.

We found that over one-quarter of children admitted to PICU with acute respiratory failure due to AVB developed PARDS according to the PALICC criteria. Half of these PARDS cases had RSV as a trigger factor.

Pneumonia has been reported as the most common precipitating cause of ARDS in children [3, 13, 14]. Acute viral bronchiolitis and viral pneumonia are overlapping, and it is often difficult to differentiate between them, particularly in children under 2 years of age. Viruses were the identified pathogen in 92% of our patients, compared to 70% in a similar study reported by Roberts et al. [5].

SV was the most common cause of PARDS in our study. It was detected in 51% of cases, as has been demonstrated in previous studies [10, 14, 15]. Similar to previous studies, patients with PARDS associated with RSV infection in our study were younger and weighed less when compared with those with PARDS caused by other viruses. We thought this may be due to age is as a major risk in RSV viral bronchiolitis. However, RSV patients had a shorter length of stay and duration of ventilation compared to children with other individual viruses [9, 16, 11]. Both the overall incidence of PARDS as well as its severity stratification were similar in those with RSV compared to children without RSV [15, 17, 18].

Comparing AVB as a trigger for PARDS with other causes of PARDS, AVB appears to have a more benign course as most cases described were mild [13, 19].

As documented in previous studies, clinical course and outcomes are worse in AVB with PARDS compared to those without PARDS [13, 19]; the mean duration of invasive mechanical ventilation was longer in children with PARDS compared to those without PARDS [4, 10, 20].

Concerning risk factors, we highlighted a significant association between bacterial co-infection and development of PARDS in our study cohort. This has also been shown in previous studies which have shown an association between bacterial co-infection, severe respiratory disease, and longer PICU stay [21]. None of the previous studies has demonstrated the association between bacterial co-infection and PARDS in AVB. This could be due to increased lung inflammation in viral/bacterial co-infection, thus making respiratory failure more severe.

Limitations to our study include those related to retrospective studies, including bias regarding patient selection as well as bias and accuracy related to the medical records.

## Conclusion

PARDS was common in critically ill children with AVB and was more common in children with RSV infection. Bacterial co-infection was significantly associated with development of PARDS, and we need to maintain a high index of suspicion of bacterial co-infection to guide antibiotic management.

Future work is needed to determine if the clinical data in this study are consistent with a common pathophysiologic mechanism leading to PARDS in children with AVB and co-infected with bacteria.

## Electronic supplementary material

ESM 1 (DOCX 18.2 kb).

## List of abbreviations

AVB: Acute viral bronchiolitis
ETT: Endotracheal tube
HMPV: Human metapneumovirus
HFOV: High frequency oscillating ventilation
LRTI: Lower respiratory tract infection
LOS: Length of stay
MV: Mechanical ventilation
NPA: Nasopharyngeal aspirate
PALICC: Pediatric Acute Lung Injury Consensus Conference
PICU: Pediatric intensive care unit
PARDS: Pediatric acute respiratory distress syndrome
PCR: Polymerase chain reaction
PaO_2_: Partial pressure of arterial oxygen
RSV: Respiratory syncytial virus
