# Supplementary material for: Acute viral bronchiolitis as a cause of pediatric acute respiratory distress syndrome
Source: Eur J Pediatr. 2020 Nov 7;180(4):1229–34. doi: 10.1007/s00431-020-03852-9 (PMC7648537; doi:10.1007/s00431-020-03852-9)
Supplement: Supplementary file 1 — (DOCX 18.2 kb). [file 431_2020_3852_MOESM1_ESM.docx]

**Supplementary Table 1: PALICC definition of PARDS.** (22)

| Age | Exclude patients with peri-natal related lung disease | | | |
| --- | --- | --- | --- | --- |
| Timing | Within 7 days of known clinical insult | | | |
| Origin of Edema | Respiratory failure not fully explained by cardiac failure or fluid overload | | | |
| Chest Imaging | Chest imaging findings of new infiltrate(s) consistent with acute pulmonary parenchymal disease | | | |
| Oxygenation | Non -invasive mechanical ventilation | Invasive mechanical ventilation | | |
|  | PARDS (No severity stratification) | Mild | Moderate | severe |
|  | Full face-mask bi-level ventilation or CPAP ≥5cm H_2_O^2^ PF ratio ≤300 SF ratio ≤264^1^ | 4≤ OI <8 5≤ OSI <7.5^1^ | 8≤ OI <16 7.5 ≤OSI <12.3^1^ | OI >16 OSI>12.3^1^ |
|  | 1-Use PaO_2_-based metric when available. If PaO_2_ is not available, wean FiO_2_ to maintain SpO_2_ ≤97% to calculate oxygen saturation index (OSI: [FiO_2_ × mean airway pressure × 100]/SpO_2_) or SpO_2_: FiO_2_ (SF) ratio.  2-For non-intubated patients treated with supplemental oxygen or nasal modes of noninvasive ventilation | | | |

**Supplementary Table 1: Pediatric acute respiratory distress syndrome (PARDS) definition** (CPAP: continuous positive airway pressure, PF ratio: partial pressure of arterial oxygen ratio to Fractional Index of Inspired Oxygen, SF ratio SpO_2_:FiO_2_, OI: oxygenation index, OSI: oxygen saturation index)

**Reference**

22. Khemani RG, Smith LS, Zimmerman JJ, Erickson S (2015) Pediatric Acute Lung Injury Consensus Conference Group. Pediatric acute respiratory distress syndrome. Pediatr Crit Care Med 16(5):23–40
